# Interactions between the predatory mite *Typhlodromalus aripo* and the entomopathogenic fungus *Neozygites tanajoae* and consequences for the suppression of their shared prey/host *Mononychellustanajoa*

**DOI:** 10.1007/s10493-012-9630-1

**Published:** 2012-10-28

**Authors:** Bonaventure Vidjannagni Agboton, Rachid Hanna, Alexis Onzo, Stefan Vidal, Andreas von Tiedemann

**Affiliations:** 1 International Institute of Tropical Agriculture, 08 BP 0932, Cotonou, Republic of Benin; 2 Division of Plant Pathology and Crop Protection, Department of Crop Sciences, University of Göttingen, Grisebachstr. 6, 37077 Göttingen, Germany; 3 Division of Agricultural Entomology, Department of Crop Sciences, University of Göttingen, Grisebachstr. 6, 37077 Göttingen, Germany

**Keywords:** Classical biological control, Predator–pathogen interactions, *Mononychellus tanajoa*, Phytoseiidae, Entomophthorales

## Abstract

The predatory mite *Typhlodromalus aripo* and the entomopathogenic fungus *Neozygites tanajoae*, both introduced from Brazil for control of the cassava green mite (CGM) *Mononychellus tanajoa*, now co-occur in cassava fields in Benin. However, studies on interactions between these two natural enemies and how they might affect CGM biological control are lacking. We determined in screenhouse experiments the effects of single and combined releases of *N. tanajoae* and *T. aripo* on CGM suppression. In the single natural enemy treatment, both *T. aripo* and *N. tanajoae* significantly reduced CGM densities, but the results of the predator (*T. aripo*) are more quickly measurable than those of the pathogen (*N. tanajoae*) in our short-term experiment. The level of CGM suppression in the combined natural enemy treatment was reduced considerably compared with *T. aripo*-alone, but only slightly when compared with *N. tanajoae* alone, with a simultaneous reduction in *T. aripo* and *N. tanajoae* abundance or prevalence. In a laboratory experiment, *T. aripo* fed more on *N. tanajoae*-infected CGM than on healthy CGM and its oviposition and survival were reduced when fed on the former compared with the latter, which can help in explaining the reduction in numbers of *T. aripo* and consequently the considerable loss in suppression of CGM in the combined natural enemy treatment in the screenhouse experiment. Together, the screenhouse and the laboratory experiments predicted negative interactions between the two natural enemies with negative consequences for CGM biological control. Long-term field observations and rigorous field experiments that simultaneously manipulate *T. aripo* and *N. tanajoae* abundance and prevalence are needed to validate the prediction of this study.

## Introduction

The relative benefits of introducing single or multiple natural enemy species for classical biological control has long been debated by ecologists and biological control practitioners (Polis et al. 1989; Sih et al. 1998; Losey and Denno 1999; Gnanvossou et al. 2003; Onzo et al. 2004; Sabelis et al. 2009). Several authors have argued for screening natural enemies and releasing only the one most effective species (Briggs 1993; Ehler and Hall 1982). This strategy has been challenged, however, by others who found little evidence that one natural enemy species provides higher suppression than two or more natural enemy species applied together (Croft et al. 1992; Huffaker et al. 1971; Lang 2003; Riechert and Lawrence 1997). Interactions among natural enemy species can have two opposite effects on target pest population (Losey and Denno 1999; Wekesa et al. 2007). Two predator species may act in a complementary fashion, thereby increasing predation risk to the prey (Heinz and Nelson 1996; Losey and Denno 1998; Onzo et al. 2004, 2005; Riechert and Lawrence 1997); or two predators may interfere with each other through intra-guild predation or some other forms of interspecific interactions, and their less-than-additive effects would reduce predation risk to the prey (Rosenheim 2001; Spiller 1986).

Interactions between natural enemies are particularly relevant to classical biological control of the cassava green mite (CGM), *Mononychellus tanajoa* (Bondar) (Acari: Tetranychidae), in Africa, because this pest is attacked by several natural enemies. Two of these, *Typhlodromalus aripo* De Leon and *Amblydromalus* (=*Typhlodromalus*) *manihoti* Moraes (Acari: Phytoseiidae), were introduced from Brazil and have established and spread widely in African cassava fields (Hanna and Toko 2003; Yaninek and Hanna 2003). Neither of these two predatory mites, however, has consistently established in the arid, semi-arid, and some subtropical mid-altitude areas (Hanna and Toko 2003; Zundel et al. 2009). Furthermore, biological control of CGM, particularly on cassava grown in the savannas and mid-altitutdes, depends on the suitability of cassava varieties for *T. aripo*, as this predator prefers to colonize cassava varieties with hairy apices, leaving those with glabrous apices vulnerable to CGM attacks (Hanna et al. 2000; Zundel et al. 2009). Together, the lack of predator persistence in some agroecologies and lack of predator colonization of glabrous varieties necessitated the search for a complementary approach to enhance CGM biocontrol in areas where it has not been fully achieved by exotic phytoseiid predators. In this regard, Brazilian isolates of the entomopathogenic fungus *Neozygites tanajoae* (Entomophthorales: Neozygitaceae), known to be virulent to CGM (Delalibera et al. 1992) and to be specific to this pest (Hountondji et al. 2002a; Agboton et al. 2009b), were introduced into Africa where they were evaluated in the laboratory and later tested in cassava fields in southern Benin (Hountondji et al. 2002b). Particularly important was the finding that *N. tanajoae* did not infect *T. aripo*, which has become the most effective exotic predator for control of CGM in Africa (Hanna and Toko 2003; Yaninek and Hanna 2003; Hanna et al. 2005). Those two exotic natural enemies of GCM on cassava in Africa, *T. aripo* and *N. tanajoae*, therefore constitute a suitable system for assessing the impact of predator–pathogen interactions on suppression of pest populations. In some regions (e.g., Benin in West Africa), *T. aripo* and *N. tanajoae* share the same cassava fields but presently data on the combined effects of this *T. aripo* and fungus on control of CGM is lacking.

The broad objective of this paper is to determine the nature of interactions between *T. aripo* and *N. tanajoae* and their consequences for CGM biological control. Specifically, we sought to determine in screenhouse experiments the relative effect of the two natural enemies, alone or together, on CGM populations over the course of at least two CGM generations. In laboratory experiments we determined the level of attack (or feeding) by *T. aripo* on *N. tanajoae*-infected or healthy CGM and consequences of such feeding on *T. aripo* survival and reproduction and how that might influence CGM biological control.

## Materials and methods

### Source of mites and fungus

Adult CGM females were obtained from a laboratory culture initiated with a cohort of eggs from CGM females collected from a cassava field free of *N. tanajoae* in southwestern Benin. Nymphs from the cultures were transferred to 2- to 3-week-old potted cassava plants in small, fine-mesh cages in a screenhouse for mass rearing. Every week, 60 live mites were mounted in 0.1 % cotton blue dissolved in lactophenol and examined with a compound microscope for the presence of fungal infections; no infection was detected in our screenhouse-reared CGM.

Adult female *T. aripo* were collected from cassava fields near the town of Sè, Department of Mono, southwestern Benin, and maintained in a laboratory at the IITA-Benin Station on a diet consisting of all stages of CGM on potted cassava plants in a screenhouse before their use in the experiments.

Isolates of *N. tanajoae* were preserved as fungus-infected, mummified CGM—referred to as “mummies”. Mummies were originally collected by R. Hanna from Alto Alegre in the state of Bahia, Brazil, in 2007 and imported to IITA-Benin facilities, where they were maintained in the laboratory at 4 °C. They were then propagated by sub-culturing them in vivo on live CGM following methods described by Hountondji et al. (2002b). For each experiment, a new batch of mummies was produced (see the next section for details) and used within 2 weeks to minimize loss of viability.

### Screenhouse experiment

An experiment was conducted on potted cassava plants in cages in a screenhouse at the Biological Control Centre for Africa, International Institute of Tropical Agriculture, Cotonou, Benin, from April to September 2007. The screenhouse (length x width x height = 24 × 8.5 × 5 m) had a canopy of Teflon plastic and sides covered with an amber screen of 32 × 32 μm mesh. To allow air circulation and to prevent excessive heat build-up, screened windows at opposite ends of the screenhouse were opened during the day. Temperatures inside the screenhouse ranged from 25 to 38 °C, and relative humidity ranged from 44 to 94 %. The experiments used 12 cages (length × width × height = 100 × 100 × 120 cm), each of which was covered with a fine, transparent mesh.

Cassava plants were obtained from farmers’ fields around Lokossa in southwestern Benin. Six cassava cuttings (20 cm long) of the variety “Agric” were planted in each of 12 plastic pots containing about 12 kg of topsoil collected from a field that had been under fallow for more than 4 years and that had not been treated with fertilizers and pesticides for at least 14 years. Pots with cassava plants were placed in the cages (one pot per cage) in the screenhouse within 48 h after planting. The cages were arranged in three groups (four cages per group with cages and pots placed on iron benches). Each group represented one replicate or block. Four treatments were randomly assigned to cages in each group. Before the experiment began, the cages were disinfested with 70 % alcohol and rinsed with distilled water.

The trial was designed as a 2 × 2 factorial experiment with the factors being predator addition or fungus addition. At the start of the experiment, each pot of plants (six plants per pot and one pot per cage) was infested with CGM by pinning two cassava leaf discs (2 cm diameter) with 25 uninfected adult female CGM on the oldest leaves of each of the six plants, for a total of 50 and 300 adult female CGM per plant and per pot respectively. Ten days later, CGM-infested plants were assigned to one of the following treatments: (1) Addition of 25 *T. aripo* adult females, (2) addition of 25 live *N. tanajoae*-exposed CGM, (3) addition of 25 *T. aripo* adult females plus 25 live *N. tanajoae*-exposed CGM, and (4) a control that remained free of predator and the fungus. The predators were placed directly on the first fully expanded leaf of each cassava plant using a camel-hair brush. The fungus was added to plants by pinning a cassava leaf disc with 25 *N. tanajoae*-infected CGM to the first fully-expanded leaf of each plant in the pot. CGM infected with *N. tanajoae* were obtained by exposing 30 healthy female CGM from the screenhouse colonies to fresh capilliconidia produced by a sporulating mummy on a 2-cm diameter leaf disc as described previously (Hountondji et al. 2002b; Agboton et al. 2009a).

Immediately prior to predator and fungus addition, one plant from each cage was removed for estimating CGM densities. This sampling was repeated on days 8, 16 and 24 after natural enemy addition. The stem of the plant was removed from the cutting, placed in a plastic bag and brought to the laboratory for counting. Eggs and active stages (larvae, nymphs, and adults) of CGM, active stages of *T. aripo* in the apex and on all the leaves, and all *N. tanajoae*-infected CGM (infected and mummified CGM) from each plant were counted in the laboratory with a binocular microscope.

### Laboratory experiment

In a laboratory experiment we determined the effect of two diets—healthy or *N. tanajoae*-infected CGM—on *T. aripo* oviposition and survival. Two cohorts of 50 gravid *T. aripo* females (6–7 days after egg hatching, 24 h-starved) were reared for use in the experiments. The experimental unit consisted of a cassava leaf disc (2 cm diameter with the abaxial surface placed up) resting on water-saturated cotton wool in an open Petri dish (13 cm diameter). One young, gravid *T. aripo* female was placed on each leaf disc and fed with 20 healthy mites or 20 infected mites per day. Each treatment was replicated 10 times, with each replicate containing both treatments. The dishes were kept in a growth chamber at 25–27 °C and at 70–90 % RH. Just before new CGM individuals were added each day, *T. aripo* eggs and CGM active stages (seemingly healthy and visibly infected) and eggs were counted on each disc and then removed before a new batch of CGM was added—to top-up the numbers at 20. The experiment continued until all predators had died; the last predator died on day 16. Daily consumption of healthy and infected mites by *T. aripo* was calculated. Because of the considerable attention needed to set-up and evaluate the treatments, all 10 replicates of each treatment were established at the same time.

### Statistical analyses

For the screenhouse experiment, the numbers of live CGM, live *T. aripo*, and *N. tanajoae*-infected and mummified mites on the leaves and in apices were summed for each plant. Means and standard errors were calculated from the sums per plant and plotted against sampling dates. We used univariate repeated measures analysis of variance with blocking to determine the effect of main treatments (i.e., *T. aripo* addition or *N. tanajoae* addition), and their interaction, and the effects of period of sampling and its interaction with the main treatments, on CGM and *T. aripo* numbers and *N. tanajoa*e prevalence. Means of CGM (actives), *T. aripo* (actives), and percent *N. tanajoae* infected CGM (infected and mummified mites) were used in statistical analyses in SAS (SAS Institute 2007). For the laboratory experiment, a *t* test was used to compare the effect of healthy vs. fungus-infect CGM on *T. aripo* oviposition. Life table analysis (SAS Institute 2007) was used to determine whether food type affected development of *T. aripo*. The effect of food type (healthy vs. fungus-infect CGM) on survival of *T. aripo* was tested using the Wilcoxon signed-rank test.

## Results

### Relative effect of *Typhlodromalus aripo* and *Neozygites tanajoae* on CGM densities in the screenhouse experiment

**Fig. 1:**
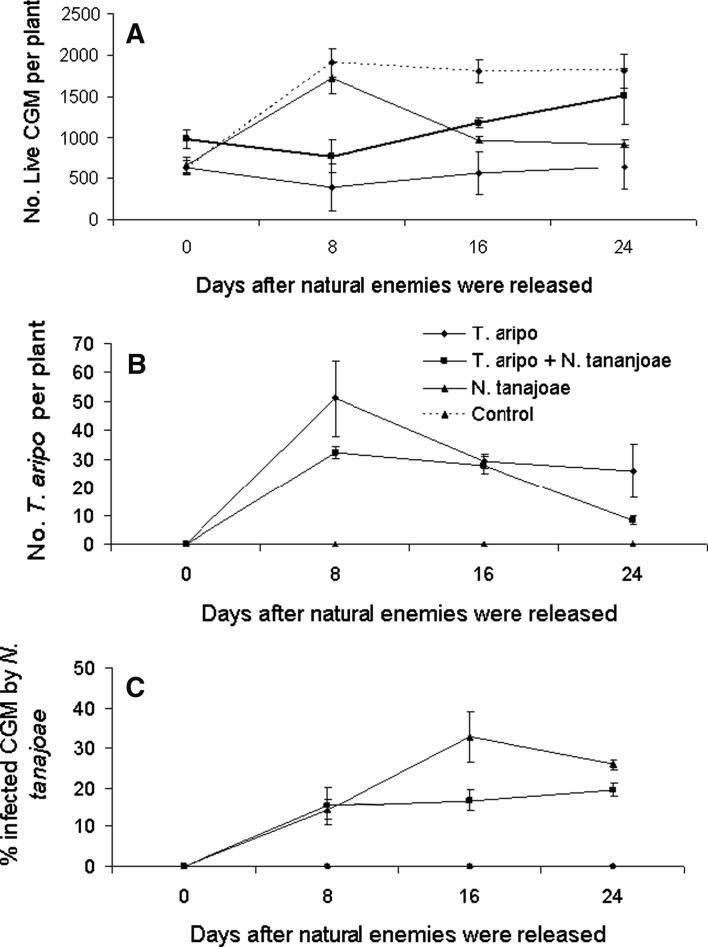
Abundance of pest mites (Cassava Green Mite: CGM), predatory mites (*Typhlodromalus aripo*), and the entomopathogenic fungus (*Neozygites tanajoae*) in the different treatments in the screenhouse experiment: **a** CGM (actives), **b** *T. aripo* (actives), and **c** *N. tanajoae* (infected and mummified mites). Numbers of CGM and *T. aripo* were log-transformed; percentages of *N. tanajoae*-infected CGM were arcsine-transformed (for normalizing the data) and averaged per plant. Day 0 is the day on which natural enemies were added

CGM densities at the time of the addition of *T. aripo* and *N. tanajoae* were similar in all treatments (2-factor ANOVA with blocking with Tukey HSD; *P* > 0.05). The addition of *T. aripo*—predator main effect—had the greatest impact on CGM numbers. In cages where this predator was added (averaged over cages with the predator alone and together with *N. tanajoae*), CGM densities were reduced by 35 % (Fig. 1a; Table 1) during the 24 days of the experiment. In contrast, the addition of *N. tanajoae*—main pathogen effect—had little effect on overall CGM densities (Fig. 1a; Table 1). This may be explained by the time take by the pathogen to develop infection and contaminate healthy mites. This is supported by the strong interaction between *T. aripo* addition and *N. tanajoae* addition. (Table 1), which necessitated the examination of simple effects for partitioning direct and indirect effects of *N. tanajoa* or *T. aripo* addition on CGM densities. We used single degrees of freedom contrasts with Bonferroni adjustment (Milliken and Johnson 1984, p. 454) to compare treatment means to the control.

**Table 1:** Repeated measures analysis of variance on mite counts and proportion of fungus-infected mites where the predatory mite *Typhlodromalus aripo* and the entomopathogenic fungus *Neozygites tanajoae* were simultaneously manipulated to determine their impact on *Mononychellus tanajoa* populations

Source of variation	*df*	*Mononychellus tanajoae*		*Typhlodromalus aripo*		*Neozygites tanajoae*	
		*F*	*P*	*F*	*P*	*F*	*P*
Between treatments^‡^							
*T. aripo* addition	1	22.4	0.003	374.7	<0.001	14.56	0.009
*N. tanajoae* addition	1	0.16	0.705	1.69	0.241	838.8	<0.001
Interaction	1	18.9	0.005	1.69	0.241	14.56	0.009
Within treatments^‡^							
Date	3	6.39	0.004	177.0	<0.001	34.0	<0.001
Date * *T. aripo* addition	3	3.61	0.033	177.0	<0.001	2.65	0.093
Date * *N. tanajoae* addition	3	0.51	0.681	2.61	0.104	34.0	<0.001
Date * *T. aripo* addition * *N. tanajoae* addition	3	10.0	<0.001	2.61	0.104	2.65	0.093

Untransformed, log-transformed and arcsine-transformed values for respectively *M. tanajoa*, *T. aripo* and *N. tanajoae* were used in the analysis

*df* degrees of freedom

^‡^Block effects had a *P* value greater than 0.05. Error *df* for between-treatment effects is 6, while error *df* for within-treatment effects were adjusted by Huynh–Feldt Epsilon where its value was <1; *M. tanajoae*: ε = 2.506; *T. aripo*: ε = 0.763; *N. tanajoae*: ε = 0.852; unadjusted error *df* = 16

The addition of *T. aripo* alone (simple effect of *T. aripo*) reduced CGM densities by 64 % (*P* < 0.05), whereas the addition of *N. tanajoae* (simple effect of *N. tanajoae*) reduced CGM densities by 30.5 % (*P* < 0.05), but the addition of both *T. aripo* and *N. tanajoae* reduced CGM densities by 27.7 % which is 36.3 % less than with *T. aripo* alone. This is a strong indication that the impact of *T. aripo* on CGM densities was considerably reduced in the presence of *N. tanajoae*, but this effect on CGM was asymmetrical as *N. tanajaoe* effect was only slightly less (9 %) when together with *T. aripo* compared with cages when it was present alone (*P* > 0.05).

In addition to main treatment and their interaction’s effects on CGM densities, there were considerable differences in the rate at which the natural enemies affected their prey/host populations over the course of the experiment. The level of effect of *T. aripo* and the interaction effect of *T. aripo* addition and *N. tanajoae* addition depended on date of sampling (*P* < 0.05 for both effects; Table 1). In contrast, the effect of *N. tanajoae* addition on CGM densities was not affected by time period during the experiment (Table 1), but this again must be interpreted in the context of the three-way interactions of the two main effects with date as this three-way interaction provides “greater sensitivity of testing the hypothesis of treatment effects and allows subplots to act as their own control to a greater extent than in only main effect analysis” (Hanna et al. 1997). Of interest are the comparisons of the effect of *T. aripo* and *N. tanajoae* on CGM densities—when the natural enemies are present alone or together—with control (i.e., absence of natural enemies).

Trends in CGM densities over the course of the experiment were different from control regardless of *T. aripo* and *N. tanajoae* being present alone or together (*F*_3,16_ = 3.24–6.65; *P* < 0.05). Stratification of the analysis by sampling date showed that in cages with *T. aripo* alone CGM densities were lower than in control cages at all three sampling dates after the start of the experiment (1-way ANOVA with blocking: *F*_1,6_ = 12.05–30.47; *P* < 0.05). CGM densities in *N. tanajoae* cages followed similar trends to those in control cages during the first 8 days (*F*_1,6_ = 0.288; *P* > 0.05) but quickly declined compared with control on the 18th day and remained at the same level on the 24th day (*F*_1,6_ = 13.83 and 6.67; *P* < 0.05). The presence of *T. aripo* and *N. tanajoae* together produced considerably different dynamics than when the two natural enemies were present alone. CGM densities quickly decreased after the addition of the two natural enemies (*F*_1,6_ = 12.59; *P* < 0.05), as in *T. aripo* cages, which indicates that this decline was largely due to *T. aripo* predation since CGM densities in *N. tanajoae* cages were similar to control. Unexpectedly, CGM densities in cages with the two natural enemies leveled-off but remained less than in the control on the 18th day (*F*_1,6_ = 5.51 and *P* < 0.05); they however increased to similar level as in the control cages by the 24th day of the experiment (*F*_1,6_ = 0.802; *P* < 0.05).

### Numbers of *Typhlodromalus aripo* and *Neozygites tanajoae* in the screenhouse experiment

We succeeded in establishing *T. aripo* in cages where this predator was released while cages that did not receive the predators remained free of them throughout the experiment (Fig. 1b; Table 1). Average *T. aripo* densities in cages where it was present alone was statistically similar to that where it was present together with *N. tanajoae* (*F*_1,6_ = 3.39 and *P* = 0.115), despite a numerical difference of 25.4 % (*T. aripo* alone: 26.41 ± 6.19 mean ± SE; *T. aripo* + *N. tanajoa*e: 17.08 ± 1.45). In both cages where it was added, *T. aripo* increased rapidly and reached slightly higher densities where it was present alone compared with its presence with *N. tanajoae*. Its densities then quickly declined and reached numerically similar levels in the two treatments with this predator, but *T. aripo* densities where it was present alone remained slightly higher than where it was present with the fungus.

As in the case of *T. aripo*, cages that did not receive *N. tanajoae* remained free of this fungus while in those that received it the fungus was able to establish and cause infections in CGM populations (Fig. 1c; Table 1). The infected CGM by *N. tanajoae* was 30 % higher where this fungus was present alone (18.22 ± 1.15 with a peak of 32.77 ± 6.21 on day 18) compared with its presence with *T. aripo* (12.88 ± 1.15 with a peak of 19.47 ± 1.79 on day 24) (*F*_1,6_ = 29.13; *P* = 0.002; Fig. 1c). Fungal infections were first detected at 8 days after the start of the experiment and were similar in the two fungus treatments (*F*_1,6_ = 0.017; *P* < 0.05; Fig. 1c). Percentage of infected CGM in cages with the fungus alone continued to increase through day 16 with a slight decline on day 24, but leveled-off during the last two sampling dates in cages where the two natural enemies were present together. The prevalence of infected CGM was higher in cages where the fungus was present alone compared with cages where it was present with *T. aripo* (day 16: *F*_1,6_ = 12.18, *P* < 0.01; day 24: *F*_1,6_ = 13.69; *P* < 0.01).

### Laboratory experiment

**Fig. 2:**
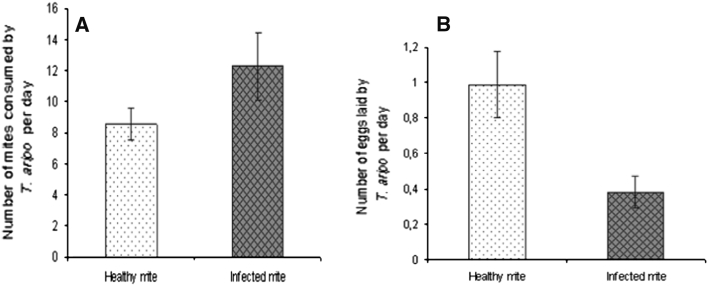
Mean (±SE) consumption of healthy CGM vs. *Neozygites tanajoae*-infected CGM by *Typhlodromalus aripo* (**a**), and the effect of diet type on *T. aripo* oviposition (**b**) in the laboratory experiment

**Fig. 3:**
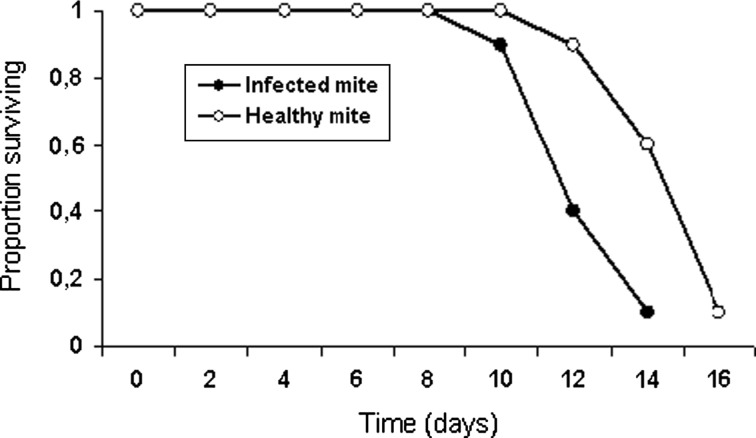
Survival curves of *Typhlodromalus aripo* feeding on *Neozygites tanajoae*-infected mites or healthy mites in the laboratory experiment

Although adult female *T. aripo* consumed both healthy and infected CGM, the predator consumed more (*P* < 0.05) infected than healthy CGM (Fig. 2a). Predator oviposition rate was also reduced (*P* < 0.05) when the predators were fed infected CGM compared with healthy CGM (Fig. 2b). The predators also lived longer (*P* < 0.01; *df* = 1; *P* = 0.0042 and 0.0048 for log-rank test and Wilcoxon test, respectively) when fed healthy CGM compared with infected CGM (Fig. 3).

## Discussion

Our screenhouse experiment confirmed the beneficial effects of both *T. aripo* and *N. tanajoae* in the biological control of cassava green mite (Alvarez Afanador et al. 1993; Roy and Pell 2000; Hountondji et al. 2002b; Hanna and Toko 2003; Yaninek and Hanna 2003; Delalibera et al. 2004; Hanna et al. 2005). When present alone, *T. aripo* numbers and *N. tanajoae* infections increased rapidly, but *T. aripo* eventually caused 2.1 fold higher level of CGM suppression compared with *N. tanajoae* (64 % for *T. aripo* and 30.5 % for *N. tanajoae*). The highest performance of *T. aripo* in our short-term screenhouse experiment is likely due to its ability to feed immediately on CGM, while the entomopathogenic fungus *N. tanajoae* requires some time to penetrate, infect, sporulate, and spread (Hajek et al. 2001). Entomopathogenic fungi kill the host only after completely growing through the host’s body (Hajek et al. 2001). It follows that transmission of infective conidia from infected to healthy hosts and the development of epizootics require both time and favorable environmental conditions (Hajek et al. 2001; Delalibera et al. 2006). In our screenhouse experiment, conditions may not have been optimal for *N. tanajoae*, and this along with the experiment’s short duration may explain why *T. aripo* performed better than *N. tanajoae* in reducing CGM numbers.

In this study, we tested the hypothesis that the presence of both natural enemies on the same cassava plant would result in additive or greater-than-additive suppression of CGM numbers, because the two natural enemies inhabit different structures of the cassava plan and therefore should not compete directly. Whereas *T. aripo* inhabits the plant apex during the day and forages on upper leaves only during the night (Onzo et al. 2003), *N. tanajoae* is restricted largely to cassava leaves (Hountondji et al. 2002b). Unexpectedly, our screenhouse experiment showed that the co-occurrence of *T. aripo* and *N. tanajoae* on the same cassava plant had a less-than-additive suppression. When present together, combined CGM suppression by *T. aripo* and *N. tanajoae* was 27.7 %, or 2.3 fold lower than when *T. aripo* was alone, and only 1.1 fold lower than when *N. tanajoae* was alone. The two natural enemies clearly interfered with each other, which negatively impacted suppression of their prey/host. This interference was asymmetric as the impact was greater on *T. aripo*’s ability to suppress CGM compared with *N. tanajoae*. It was clear that the presence of *T. aripo* decreased CGM infections by *N. tanajoa* by 30 % (*F*_1,6_ = 29.13; *P* = 0.002). Similarly, the presence of *N. tanajoae* reduced *T. aripo* numbers by 25.2 %, but this difference was not significant (*F*_1,6_ = 3.39 and *P* = 0.115), probably due to large variations in *T. aripo* numbers on day 8 and 14 (51.0 ± 13.05 and 25.7 ± 9.49 respectively) in cages where *T. aripo* was alone Similarly, cages that did not receive *N. tanajoae* remained free of this fungus while in those that received it the fungus was able to establish and cause infections in CGM populations (Fig. 1c; Table 1). Statistical similarities or differences notwithstanding, both natural enemies reduced each other’s numbers/prevalence by nearly equal level, which evidently caused the reduction in CGM suppression in the combined presence of the two natural enemies. Undoubtedly, however, the negative impact of *N. tanajoae* on *T. aripo* is the likely cause of this substantial loss in CGM suppression, as *T. aripo* alone was able to reduce CGM populations by 2.1 fold more than *N. tanajoe* alone. Our laboratory evidence and that by Ariori and Dara (2007) provides possible explanations for the observed interactions between *T. aripo* and *N. tanajoae* and consequences for the suppression of their shared prey/host the cassava green mite.

The reduction in *T. aripo* abundance in the combined treatment of the screenhouse experiment was at least partially explained by the laboratory experiment that used cassava leaf discs on which *T. aripo* females were fed either *N. tanajoae*-infected CGM or healthy CGM. Those laboratory data indicated that *T. aripo* consumed significantly more infected than healthy CGM, perhaps because infected mites are less mobile and thus more easily captured than healthy mites. By feeding on infected mites, *T. aripo* not only reduced the density of fungal inoculum but also reduced its own reproduction and longevity, probably because of the poorer nutritional quality of infected compared with healthy mites, or together with only partial consumption of infected mites (particularly if the greater attack on the infected mites was due to greater prey catch efficiency of the predator rather than preference for the infected mites). It is also possible that *N. tanajoae* produces toxins, like some entomopathogenic fungi (Boucias and Pendland 1998) that could harm a predator that consumed the infected CGM.

Ariori and Dara (2007) demonstrated that the consumption of pathogen-infected pest mites by predatory mites can reduce the effectiveness of the pathogen. This effect may be enhanced by a ‘preference’ of the predator for feeding on infected hosts, thereby reducing the entomopathogen inoculum, which otherwise would be dispersed among living host mites. The greater consumption of infected mites, however, may not mean a preference for these mites by the predator, but rather a greater prey catch efficiency by the predator on the relatively less mobile infected mites. Nevertheless, the interactions reported by Ariori and Dara (2007) is consistent with our results in that high *T. aripo* densities in the combined treatments were associated with decreased *N. tanajoae* infections. This mechanism may explain also the failure of *N. tanajoae* and the predatory mite *Neoseiulus idaeus* to control a population of CGM in central Bahia, Brazil (Elliot et al. 2008). While predation of *T. aripo* on infected CGM may reduce the level of fungus inoculum, the predator could also indirectly increase fungus inoculum by spreading fungus propagules as it foraged on cassava plants. This effect could be greater in the field than in our screenhouse experiment.

Taken together, the results of our screenhouse and laboratory experiments point to interactions between *T. aripo* and *N. tanajoae* that might affect the dynamics of these natural enemies in the field and in turn their impact on the target host mite *M. tanajoa*. Our experimental results are not sufficient, however, to declare that the presence of the two predators together will have less effect on CGM populations compared with their presence alone. Cassava plants used in our screenhouse experiment were young (four to eight weeks old) and small relative to the 12 month typical growth cycle (from planting to harvest) and large size of cassava plants in the field in West Africa. Larger plants may facilitate the dispersal of prey to avoid predators (Magalhães et al. 2002) and pathogens (Hountondji 2005), which may result in greater coexistence of the two natural enemies. Furthermore, cassava plants in cages in the screenhouse tended to have smaller apices than equally-aged plants in the field; this together with the small plant size and shading in the screenhouse results in *T. aripo* being present on cassava leaves more often than under field conditions, which probably increases the intensity of the interactions between the two natural enemies. Similar results were obtained in screenhouse experiments by Onzo et al. (2004) in which significant asymmetrical negative interactions occurred between *T. aripo* and another leaf inhabiting mite *A. manihoti*. In subsequent field observations (Onzo et al. 2003; Zannou et al. 2007) and field manipulative experiments (A. Onzo, R. Hanna, and M. W. Sabelis, unpubl. data), no such negative interactions were observed. Furthermore, all field observations and experimental manipulations showed that the presence of two predators is more beneficial for biological pest control than when each predator is alone, despite the negative inter-predator interactions observed in the screenhouse. Similar field experiments are needed in which *T. aripo* abundance and *N. tanajoae* incidence are simultaneously manipulated to determine their relative effects—alone or in combination—on CGM populations.

The results of our experiments underscore the complexity and difficulty we and countless others have faced in deciding whether to release one or multiple natural enemies, particularly where natural enemies interact in a way that will either reduce or enhance the suppression of the target pest(s), which largely depends on the characteristics of the natural enemies and the ecosystem. The debate on the use of single ore multiple natural enemy species in biological control of arthropod pests will surely continue, but with every carefully planned and executed study we will move closer to a unified framework for predicting which system would benefit from a particular release strategy. Until then, there is no substitute for field experiments that simultaneously manipulate the most significant explanatory variables to determine their outcome in the suppression of the target pest.
